# The influence of APOE status on rate of cognitive decline

**DOI:** 10.1007/s11357-024-01069-4

**Published:** 2024-01-22

**Authors:** Cassandra Morrison, Michael D. Oliver, Virginia Berry, Farooq Kamal, Mahsa Dadar

**Affiliations:** 1 Department of Psychology, Carleton University, Ontario, K1S 5B6 Canada; 2 Department of Psychological Science and Neuroscience, Belmont University, Nashville, TN 37212 USA; 3 Belmont Data Collaborative, Belmont University, Nashville, TN 37212 USA; 4 Department of Psychiatry, McGill University, Montreal, QC H3A 1A1 Canada; 5 Douglas Mental Health University Institute, Montreal, QC H4H 1R3 Canada

**Keywords:** Subjective cognitive decline, APOE status, Cognitive change, Mild cognitive impairment, Cognitively unimpaired older adults

## Abstract

**Background:**

Apolipoprotein (APOE) ɛ4 positivity and subjective cognitive decline (SCD) both increase risk of Alzheimer’s disease (AD) development. However, few studies have examined the relationship between SCD and APOE status, especially using longitudinal data. The current study examined whether APOE is associated with the rate of cognitive change in SCD and mild cognitive impairment (MCI).

**Methods:**

A sample of 3494 older adults (1990 normal controls, NC, 775 SCD, and 729 MCI) with a mean follow-up of 9.09 years were included from the Rush Alzheimer’s Disease Center Research Sharing Hub. Linear mixed effects models examined the relationship between APOE status and cognitive change in older adults with SCD normal controls, and people with MCI.

**Results:**

The presence of at least one ɛ2 allele in SCD and MCI results in cognitive change rates similar to a NC with the ɛ3ɛ3 genotype. Older adult SCD-ɛ4 individuals exhibited increased rate of cognitive decline compared to all groups, including NC-ɛ4 and MCI-ɛ4.

**Conclusion:**

People with SCD with at least one ɛ4 allele experience increased rates of cognitive decline compared to cognitively healthy older adults and people with MCI. These findings have important implications for treatments and interventions and can improve future research and clinical trials by targeting people in the preclinical AD phase (i.e., SCD) who also possess at least one APOE ɛ4 allele.

**Supplementary information:**

The online version contains supplementary material available at 10.1007/s11357-024-01069-4.

## Introduction

Affecting nearly 55 million people worldwide, Alzheimer’s disease (AD) is by far the most common cause of dementia [1]. Functionally, AD is characterized by a progressive decline in memory and other cognitive abilities, resulting in the inability to perform normal activities of daily living [2]. People who exhibit deficits in cognitive functioning but maintain the ability to perform daily life activities independently, may be at a midpoint between healthy cognitive aging and dementia and are categorized as having mild cognitive impairment (MCI) [2, 3]. The structural brain changes that result in these functional declines have been suggested to occur nearly 20 years prior to the onset of clinical symptoms [4]. This knowledge has led to a plethora of research targeting factors associated with increased risk and subsequent development of AD.

Several risk factors have been identified to be associated with AD. One of the most significant risk factors is a genetic factor from the variant of the Apolipoprotein E (APOE) gene. Those who have an APOE ε4 allele have an increased risk of AD [5]. Although the presence of an ɛ4 allele does not necessarily imply that one will develop AD, studies have revealed that heterozygous ε4 carriers have a 3–4 times higher risk, and homozygous ε4 carriers have an 8–12 times increased risk for AD development compared to non-carriers [6]. Research has also shown that individuals who are ɛ4 positive have significantly different trajectories in the clinical manifestation and pathogenesis of disease. For example, ɛ4 carriers exhibit an earlier age of AD onset [5] and increased rates of cognitive decline [7] compared to those who are not ɛ4 carriers. On the other hand, the ɛ2 allele has been shown to offer protective effects against risk of AD [8], while ɛ3 does not offer any protective or detrimental effects towards AD [9].

Another factor that increases risk of MCI [10], AD development [11, 12], and cognitive decline [11] is subjective cognitive decline (SCD). SCD is self-reported cognitive decline in older adults with no objective evidence of cognitive decline [11]. Some research has suggested that compared to cognitively healthy older adults without SCD (normal controls—NC), those with SCD show higher frequencies of APOE ε4 positivity [13]. However, a systematic review observed that ɛ4 frequency does not differ between cognitively healthy older adults with and without SCD [14]. This review observed that both APOE positivity and SCD provide individual and multiplicative risks towards cognitive decline. While they indicate that SCD status and APOE ɛ4 positivity increase risk of developing objective cognitive impairment, they note that a limited understanding of the relationship between SCD and APOE status remains, especially given the paucity of studies that have employed longitudinal cohorts [14].

This paper was designed to expand on the current understanding of the relationship between SCD and APOE status. To do so, we compared different APOE profiles in cognitively healthy older adults with and without SCD and people with MCI. This study design allowed us to examine the rate of cognitive change in people with SCD and whether APOE status influences the rate of change in this group compared to cognitively healthy older adults without SCD and people with MCI. The main goal was to determine if APOE status would influence the rate of cognitive change in people with SCD and MCI. That is, do people with SCD and MCI who have an ε2 allele have similar rates of cognitive decline to cognitively healthy older adults, and do people with SCD who have at least one ε4 allele have increased rates of cognitive decline compared to cognitively older adults and people with MCI with at least one ε4 allele.

## Methods

### Participants

Data used in preparation of this article were obtained from the RADC Research Resource Sharing Hub (www.radc.rush.edu). Participants provided informed written consent to participate in one of three cohort studies on aging and dementia: 1) Minority Aging Research Study (MARS) [15], 2) Rush Alzheimer's Disease Center African American Clinical Core (RADC AA Core) [16], or 3) the Rush Memory and Aging Project (MAP) [17]. Detailed information on each cohort included in this study can be found in Supplementary Table 1. Further information can also obtained at https://www.radc.rush.edu/docs/parentStudyDesigns.htm.

Participant inclusion criteria for this specific study were as follows: 1) either cognitively healthy status or MCI status at baseline, 2) had at least two cognitive assessments, 3) were at least 55 years of age at baseline, and 4) had APOE genotyping completed. For all cohorts, a clinical diagnosis of cognitive status (normal control or MCI) was completed using a three-stage process including computer scoring of cognitive tests, clinical judgment by a neuropsychologist, and diagnostic classification by a clinician based on criteria of the joint working group of the National Institute on Aging and the Alzheimer’s Association (NIA-AA) [18]. For cognitively healthy older adults to be included they must have also completed the questionnaire assessing subjective memory complaints. This questionnaire allowed for the cognitively health older adults to be classified as normal control or subjective cognitive decline.

Subjective cognitive decline was evaluated in cognitively healthy older adults based on two questions examining memory complaints. Participants were asked, “About how often do you have trouble remembering things?” and “Compared to 10 years ago, would you say that your memory is much worse, a little worse, the same, a little better, or much better?”. Both questions were scored using a scale of 1 to 5 with 1 being never/much better and 5 being often/worse. Following past research and the Rush recommendations, if the participants had a composite score of 8–10 on these two questions they were classified as having memory complaints [19]; reported as subjective cognitive decline (SCD), and if they had a composite score < 8, they were classified as normal controls in this paper.

Participants were divided into one of three groups [cognitively normal (NC), subjective cognitive decline (SCD), and mild cognitive impairment (MCI)] and one of three APOE profiles [ε2 = ε2ε2 or ε2ε3; ε3 = ε3ε3; or ε4 = ε4ε4 or ε4ε3]. Due to limited sample sizes of the homozygous ε2 and ε4 groups, we combined them with the heterozygous ε2ε3 and ε4ε3 groups respectively. Additionally, the focus of the paper was to compare individuals with ε2 to ε4, so those with the ε2ε4 genotype were excluded from analysis. To ensure group differences in follow-up duration did not impact the results, participants from each group were matched based on follow-up year. Additionally, sex, age, and education levels were included in the models as covariates to ensure results were not driven by these demographic factors. A total of 3494 older adults (NC = 1990, SCD = 775, MCI = 729), with a mean follow-up of 9.09 years (and a total of 33,722 follow-up timepoints) were included in this study. Participants had annual follow-ups in which they completed the cognitive testing. Participant demographic information by group is presented in Table 1.

**Table 1:** Participant demographic information by group

	NC-ε2	NC-ε3	NC-ε4	SCD-ε2	SCD-ε3	SCD-ε4	MCI-ε2	MCI-ε3	MCI-ε4
Sample Size	288	1267	435	112	477	189	93	406	230
Age (years)	75.8 ± 7.39	74.0 ± 6.83	75.6 ± 7.26	76.7 ± 8.34	74.9 ± 7.30	77.1 ± 7.15	80.2 ± 8.04	77.3 ± 7.13	79.8 ± 7.53
Sex (females)	217 (75%)	957 (76%)	348 (80)	78 (70%)	140 (74%)	366 (77%)	63 (68%)	292 (72%)	168 (72%)
Education (years)	15.8 ± 3.49	16.2 ± 3.91	15.9 ± 3.93	15.4 ± 3.75	16.0 ± 3.62	15.6 ± 3.62	15.6 ± 3.28	15.9 ± 3.55	15.5 ± 3.73
Follow-up timepoints	2811	12,589	4383	1216	4570	1680	792	3536	2126
Mean follow-up (years)	9.10 ± 5.61	9.25 ± 5.54	9.37 ± 5.36	10.2 ± 5.07	8.86 ± 5.38	8.47 ± 5.11	8.01 ± 4.43	7.98 ± 4.40	8.66 ± 4.63
Median follow-up (years)	8	8	9	7	7	8	9	8	7
Follow-up minimum and maximum (years)	0–27	0–27	0–27	2–26	1–24	0–26	0–25	1–26	1–26
Convertors	79 (27%)	427 (34%)	162 (37%)	47 (42%)	199 (42%)	87 (47%)	28 (30%)	171 (42%)	136 (59%)

Numbers are presented as total (percentage of group) or mean ± standard deviation. NC = cognitively healthy older adults without subjective cognitive decline. SCD = cognitively healthy older adults with subjective cognitive decline. MCI = mild cognitive impairment. NC and SCD was younger than MCI. MCI had increased ε4 and decreased ε3 frequency compared to NC

### Cognitive scores

A neurological battery comprising 19 cognitive assessments was administered to all participants annually [15]. Five cognitive domains were assessed through the selected 19 tests: episodic memory, semantic memory, working memory, visuospatial ability, and perceptual speed. Episodic memory was assessed through scores from Word List Memory, Word List Recall, Word List Recognition, and immediate and delayed recall scores of both Story A on Logical Memory and the East Boston Story. Semantic memory was assessed through performance on Verbal Fluency, and on both a 15-item reading test, and a version of the Boston Naming Test. The working memory domain was measured through performance on Digit ordering, as well as Digit Span Forwards and Backwards. Visuospatial ability was assessed through performance on a 16-item version of the Progressive Matrices and a 15-item version of Judgement and Line Orientation. Perceptual speed was measured through performance on Number Comparison, two indices from a modified version of the Stroop Neuropsychological Screening Test, and the Symbol Digit Modalities Test. Raw scores on each of the individual tests were converted to z-scores using the baseline mean (SD); and the z-scores of the tests from each domain were then averaged. An individual’s standard performance across all 19 of these tests was averaged to create a measure of global cognitive function [20]. More information for the specific tests used for each category can be obtained from https://www.radc.rush.edu/.

### Statistical analysis

Analyses were performed using ‘R’ software version 4.0.5. Linear mixed effects models were used to investigate the association between each group (NCε2, NCε3, NCε4, SCDε2, SCDε3, SCDε4, MCIε2, MCIε3, and MCIε4) and rate of cognitive change for each cognitive domain (global cognition, episodic memory, semantic memory, perceptual orientation, perceptual speed, and working memory). All continuous values (except follow-up year) were z-scored within the population prior to analyses. Follow-up year was not z-scored to allow for the calculation of annual rate of change. All results were corrected for multiple comparisons using false discovery rate (FDR) of 0.05, p-values are reported as raw values with significance then determined by FDR correction [21].

The first analysis was completed within each group individually (NC, SCD, and MCI separately) to determine within group effects of APOE status. The interaction of interest was TimeFromBaseline:Group to determine if rate of cognitive decline differed within each APOE status (i.e., ε2, ε3, ε4) in each group. In this analysis, Participant ID was included as a categorical random effect to account for repeated measures of the same participant. Baseline age (Age_bl), sex, years of education, TimeFromBaseline, and Group were included as covariates in all models.

The second analysis used the same model but included SCD, NC, and MCI in the same model. This analysis was used to allow for comparison of the impact of APOE status on rate of cognitive decline in people with SCD to that of the other groups. The interaction of interest was TimeFromBaseline:Group to estimate the annual rate of cognitive decline in each group and determine if cognitive change over time differed for SCD groups compared to NC and MCI. Participant ID was included as a categorical random effect to account for repeated measures of the same participant. Baseline age (Age_bl), sex, years of education, TimeFromBaseline, and Group were included as covariates in all models.

1 $$CognitiveScore \sim Age\_bl+ Sex + Education + TimeFromBaseline + Group +TimeFromBaseline:Group + (1 | ID)$$

It should be noted that timepoints with missing or invalid cognitive tests were rare across all batteries (1.48% for semantic memory, 1.98% for working memory, 2.07% for global cognition, 8.03% for episodic memory, 9.89% for perceptual orientation, and 14.24% for perceptual speed). The annual rate of change was calculated for each group based on the estimated slope for each group and cognitive domain. Cognitive status at 20 years of follow up was also calculated using the model estimates [i.e., intercept + (slope*year) with the value for year being 20].

## Results

### Within group impact of APOE on rate of cognitive decline

Table 2 presents the TimeFromBaseline:Group interaction estimates from the linear mixed effects model examining the influence of APOE status on rate of cognitive decline within each group (NC, SCD, and MCI) individually.

For the NC, people with ε2 had a slower rate of cognitive decline compared to those with ε4 and ε3 for all cognitive domains [*t* belongs to (-10.50 – -2.34), *p* < 0.05] except for perceptual orientation in those with an ε3 (*p* > 0.05). Furthermore, people with ε3 had a slower rate of cognitive decline compared to ε4 in all domains [*t* belongs to (-8.33 – -3.29), *p* < 0.005]. For the SCD group, people with ε2 had a slower rate of cognitive decline than ε3 in global cognition, semantic memory, and working memory [*t* belongs to (-2.93 – -3.99), *p* < 0.005], and a slower rate of change than people with ε4 in global cognition, episodic memory, perceptual orientation, semantic memory, and working memory [*t* belongs to (-8.91 – -2.49), *p* < 0.05]. For the MCI group, people with ε2 did not have a rate of cognitive decline that differed from those with an ε3 in any cognitive domain. Those with an ε2 and ε3 had a slower rate of cognitive decline than ε4 in global cognition, episodic memory, and semantic memory [*t* belongs to (-4.35 – -2.29), *p* < 0.05].

**Table 2:** Linear mixed effects model outputs showing the TimeFromBaseline:Group interaction for each group separately

Within Group Effects	Global	Episodic Memory	Perceptual Orientation	Perceptual Speed	Semantic Memory	Working Memory
NCε2:NCε3	**β = -0.07,** ***t*** **=** ***-*** **5.20,** ***p*** **< 0.001**	**β = -0.06,** ***t*** **= -3.92,** ***p*** **< 0.001**	β = -0.01, *t* = -0.43, *p* = 0.66	**β = -0.04,** ***t*** **= -3.48,** ***p*** **< 0.001**	**β = -0.05,** ***t*** **= -3.41,** ***p*** **< 0.001**	**β = -0.03,** ***t*** **= -2.34,** ***p*** **= 0.019**
NCε2:NCε4	**β = -0.07,** ***t*** **=** ***-*** **10.50,** ***p*** **< 0.001**	**β = -0.12,** ***t*** **=** ***-*** **7.89,** ***p*** **< 0.001**	**β = -0.05,** ***t*** **= -2.75,** ***p*** **= 0.005**	**β = -0.09,** ***t*** **= -6.19,** ***p*** **< 0.001**	**β = -0.12,** ***t*** **= -7.84,** ***p*** **< 0.001**	**β = -0.09,** ***t*** **= -5.78,** ***p*** **< 0.001**
NCε3:NCε4	**β = -0.09,** ***t*** **=** ***-*** **8.30,** ***p*** **< 0.001**	**β = -0.07,** ***t*** **= -6.19,** ***p*** **< 0.001**	**β = -0.04,** ***t*** **= -3.29,** ***p*** **= 0.001**	**β = -0.05,** ***t*** **= -4.40,** ***p*** **= 0.001**	**β = -0.07,** ***t*** **= -6.77,** ***p*** **< 0.001**	**β = -0.06,** ***t*** **= -5.18,** ***p*** **< 0.001**
SCDε2:SCDε3	**β = -0.06,** ***t*** **=** ***-*** **2.93,** ***p*** **= 0.002**	β = -0.02, *t* = -0.81, *p* = 0.42	β = -0.03, *t* = -1.27, *p* = 0.20	β = -0.02, *t* = -2.15, *p* = 0.031	**β = -0.09,** ***t*** **=** ***-*** **3.99,** ***p*** **< 0.001**	**β = -0.07,** ***t*** **=** ***-*** **3.03,** ***p*** **= 0.001**
SCDε2:SCDε4	**β = -0.24,** ***t*** **=** ***-*** **8.91,** ***p*** **< 0.001**	**β = -0.18,** ***t*** **=** ***-*** **6.50,** ***p*** **< 0.001**	**β = -0.07,** ***t*** **= -2.49,** ***p*** **= 0.013**	β = -0.05, *t* = -1.01, *p* = 0.31	**β = -0.21,** ***t*** **=** ***-*** **7.65,** ***p*** **< 0.001**	**β = -0.20,** ***t*** **=** ***-*** **7.17,** ***p*** **< 0.001**
SCDε3:SCDε4	**β = -0.17,** ***t*** **=** ***-*** **8.30,** ***p*** **< 0.001**	**β = -0.16,** ***t*** **=** ***-*** **7.48,** ***p*** **< 0.001**	β = -0.04, *t* = -1.84, *p* = 0.07	β = -0.03, *t* = -1.68, *p* = 0.09	**β = -0.12,** ***t*** **=** ***-*** **5.55,** ***p*** **< 0.001**	**β = -0.13,** ***t*** **=** ***-*** **5.94,** ***p*** **< 0.001**
MCIε2:MCIε3	β = -0.05, *t* = -1.38, *p* = 0.17	β = -0.01, *t* = -0.27, *p* = 0.79	β = -0.06, *t* = -1.48, *p* = 0.14	β = -0.01, *t* = -0.11, *p* = 0.91	β = -0.04, *t* = -1.14, *p* = 0.25	β = -0.06, *t* = -1.64, *p* = 0.10
MCIε2:MCIε4	**β = -0.12,** ***t*** **=** ***-*** **3.06,** ***p*** **= 0.001**	**β = -0.09,** ***t*** **=** ***-*** **2.29,** ***p*** **= 0.022**	β = -0.07, *t* = -1.67, *p* = 0.09	β = 0.01, *t* = 0.29, *p* = 0.77	**β = -0.16,** ***t*** **=** ***-*** **3.90,** ***p*** **< 0.001**	β = -0.09, *t* = *-*2.19, *p* = 0.028
MCIε3:MCIε4	**β = -0.07,** ***t*** **=** ***-*** **2.69,** ***p*** **= 0.007**	**β = -0.05,** ***t*** **=** ***-*** **3.15,** ***p*** **= 0.001**	β = -0.01, *t* = -0.39, *p* = 0.70	β = 0.01, *t* = 0.62, *p* = 0.54	**β = -0.11,** ***t*** **=** ***-*** **4.35,** ***p*** **< 0.001**	β = -0.02, *t* = *-*0.97, *p* = 0.33

NC = cognitively healthy older adults without subjective cognitive decline. SCD = cognitively healthy older adults with subjective cognitive decline. MCI = mild cognitive impairment. ε2 = someone with either ε2ε3 or ε2ε2. ε3 = someone with ε3ε3. ε4 = someone with ε4ε3 or ε4ε4. Bold values are those that remained significant after correction for multiple comparisons

### Between group impact of APOE status on rate of cognitive decline

**Fig. 1:**
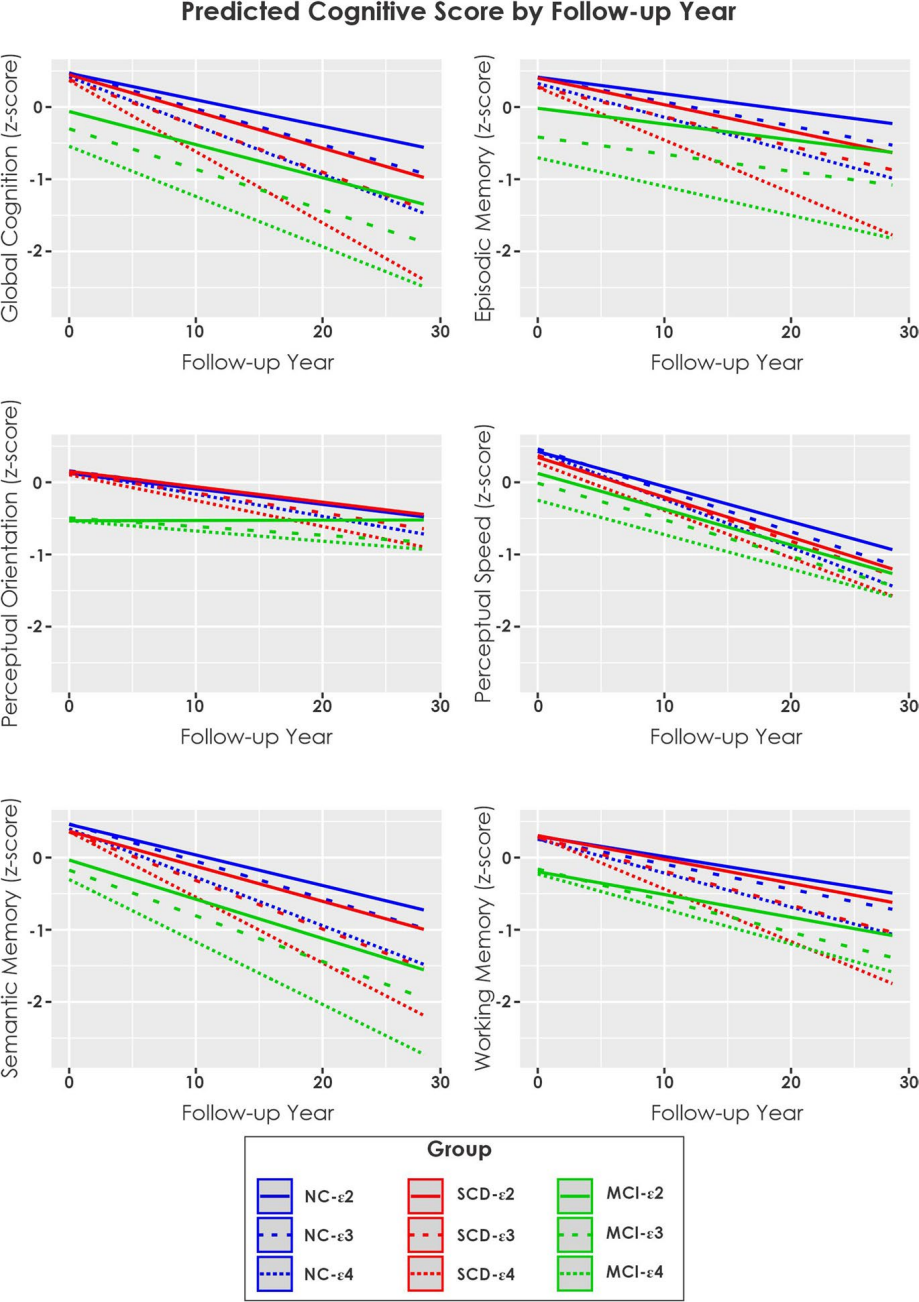
Estimated longitudinal cognitive change over time by group and cognitive domain. Notes: NC = cognitively normal controls. SCD = subjective cognitive decline. MCI = mild cognitive impairment

Table 3 presents the TimeFromBaseline:Group interaction examining between group effects on rate of change for each cognitive domain. Table 4 presents the annual rate of change for each group by APOE status. The bolded values represent the three groups with the largest annual rate of change and the italicized represent the three groups with the lowest cognitive score at 20 years. Figure 1 shows predicted longitudinal cognitive change over time by group and cognitive domain. Supplementary Fig. 1 shows the uncorrected longitudinal cognitive change over time by group and cognitive domain.

Older adults with SCD-ε2 exhibited a slower rate of cognitive decline compared to NC-ε4 in all cognitive domains [*t* belongs to (-2.22 – -4.02), *p* < 0.05], except perceptual orientation in which their rate of change did not differ. Additionally, MCI-ε2 also exhibited slower rates of cognitive decline compared to NC-ε4 in all cognitive domains [*t* belongs to (-2.45 – -4.72), *p* < 0.05], except semantic memory in which their rate of change did not differ. Both SCD-ε2 and MCI-ε2 did not differ from NC-ε3 in any cognitive domain (*p* < 0.05) except MCI-ε2 exhibited a slower rate of change in perceptual orientation than NC-ε3 (*t* = *-*3.65, *p* < 0.001). That is, both people who have SCD and MCI who also have at least one ε2 (i.e., ε2ε2 or ε2ε3) have a lower annual rate of cognitive change compared to cognitively healthy older adults with at least one ε4 (i.e., ε4ε3 or ε4ε4) and similar rates of change to cognitively healthy older adults with the ε3ε3 genotype.

Older adults with SCD-ε4 exhibited increased rates of cognitive change compared to NC-ε4 in all cognitive domains [*t* belongs to (5.54 – 7.74), *p* < 0.001], except perceptual orientation and perceptual speed. Furthermore, this group (SCD-ε4) also exhibited increased rates of cognitive change compared to MCI-ε4 in all domains [*t* belongs to (3.98 – 6.74), *p* < 0.001] except semantic memory. That is, people in the SCD-ε4 had increased rates of annual change compared to all other ε4 groups except in a select few domains.

**Table 3:** Linear mixed effects model outputs for analysis 2 showing the TimeFromBaseline:Group interaction examining between-group effects

Between Group Effects	Global	Episodic Memory	Perceptual Orientation	Perceptual Speed	Semantic Memory	Working Memory
NCε4:SCDε2	**β = -0.02,** ***t*** **=** ***-*** **3.85,** ***p*** **< 0.001**	**β = -0.01,** ***t*** **=** ***-*** **2.22,** ***p*** **= 0.025**	β = -0.01, *t* = *-*1.95, *p* = 0.052	**β = -0.01,** ***t*** **=** ***-*** **2.75,** ***p*** **= 0.005**	**β = -0.02,** ***t*** **=** ***-*** **4.02,** ***p*** **< 0.001**	**β = -0.01,** ***t*** **=** ***-*** **3.06,** ***p*** **= 0.022**
NCε3:SCDε2	β** < **0.01, *t* = 0.25, *p* = 0.71	β** < **0.01, *t* = 0.89, *p* = 0.37	β** < -**0.01, *t* = *-*0.31, *p* = 0.75	β** < -**0.01, *t* = *-*0.53, *p* = 0.60	β** < -**0.01, *t* = *-*0.71, *p* = 0.48	β** < -**0.01, *t* = *-*0.40, *p* = 0.69
NCε4:MCIε2	**β = -0.02,** ***t*** **=** ***-*** **3.65,** ***p*** **< 0.001**	**β = -0.03,** ***t*** **=** ***-*** **4.08,** ***p*** **< 0.001**	**β = -0.03,** ***t*** **=** ***-*** **4.72,** ***p*** **< 0.001**	**β = -0.03,** ***t*** **=** ***-*** **3.01** ***p*** **= 0.003**	β = -0.01, *t* = *-*2.03, *p* = 0.043	**β = -0.02,** ***t*** **=** ***-*** **2.45,** ***p*** **= 0.014**
NCε3:MCIε2	β** < -**0.01, *t* = *-*0.69, *p* = 0.49	β** < -**0.01, *t* = *-*1.93, *p* = 0.053	**β = -0.02,** ***t*** **=** ***-*** **3.65,** ***p*** **< 0.001**	β** < -**0.01, *t* = *-*1.42, *p* = 0.16	β** < **-0.01, *t* = -0.45, *p* = 0.65	β** < -**0.01, *t* = *-*0.52, *p* = 0.60
NCε4:SCDε4	**β = 0.03,** ***t*** **= 7.74,** ***p*** **< 0.001**	**β = 0.03,** ***t*** **= 6.26,** ***p*** **< 0.001**	β** < **0.01, *t* = 1.13, *p* = 0.29	β** < **-0.01, *t* = -0.23, *p* = 0.82	**β = 0.02,** ***t*** **= 5.54,** ***p*** **< 0.001**	**β = 0.03,** ***t*** **= 6.00,** ***p*** **< 0.001**
MCIε4:SCDε4	**β = 0.03,** ***t*** **= 6.16,** ***p*** **< 0.001**	**β = 0.03,** ***t*** **= 6.74,** ***p*** **< 0.001**	**β = 0.02,** ***t*** **= 4.09,** ***p*** **< 0.001**	**β = 0.02,** ***t*** **= 3.98,** ***p*** **< 0.001**	β** < **0.01, *t* = 0.89, *p* = 0.38	**β = 0.02,** ***t*** **= 4.82,** ***p*** **< 0.001**

NC = cognitively healthy older adults without subjective cognitive decline. SCD = cognitively healthy older adults with subjective cognitive decline. MCI = mild cognitive impairment. ε2 = someone with either ε2ε3 or ε2ε2. ε3 = someone with ε3ε3. ε4 = someone with ε4ε3 or ε4ε4. Bold values are those that remained significant after correction for multiple comparisons

**Table 4:** Annual rate of change by Group and cognition at 20 years

Group	Global		Episodic Memory		Perceptual Orientation		Perceptual Speed		Semantic Memory		Working Memory	
	Annual	20 years	Annual	20 years	Annual	20 years	Annual	20 years	Annual	20 years	Annual	20 years
NCε2	-0.037	-0.34	-0.020	-0.01	-0.022	-0.33	-0.051	-0.57	-0.030	-0.20	-0.028	-0.26
NCε3	-0.050	-0.60	-0.030	-0.20	-0.023	-0.32	-0.060	-0.71	-0.050	-0.55	-0.035	-0.45
NCε4	**-0.067**	-0.94	**-0.044**	-0.58	**-0.031**	-0.49	**-0.069**	-0.93	**-0.066**	-0.96	**-0.047**	-0.69
SCDε2	-0.051	-0.62	-0.034	-0.28	-0.022	-0.30	**-0.062**	-0.87	-0.047	-0.59	-0.033	-0.36
SCDε3	-0.064	-0.93	**-0.038**	-0.51	**-0.028**	-0.44	**-0.062**	-0.89	-0.066	-0.97	**-0.047**	-0.64
SCDε4	**-0.099**	*-1.63*	**-0.070**	*-1.15*	**-0.036**	*-0.61*	**-0.068**	*-1.10*	**-0.090**	*-1.45*	**-0.075**	*-1.20*
MCIε2	-0.054	-0.98	-0.019	-0.42	0.00	-0.55	-0.052	-0.92	-0.053	-1.16	-0.032	-0.84
MCIε3	-0.056	*-1.42*	-0.021	*-0.78*	-0.012	*-0.74*	-0.053	*-1.04*	-0.060	*-1.4* *0*	-0.044	*-1.03*
MCIε4	**-0.070**	*-2.00*	-0.037	*-1.47*	-0.014	*-0.83*	-0.050	*-1.25*	**-0.085**	*-2.05*	**-0.049**	*-1.23*

SCD = Subjective cognitive decline. NC = Normal Controls. MCI = mild cognitive impairment. ε2 = someone with either ε2ε3 or ε2ε2. ε3 = someone with ε3ε3. ε4 = someone with ε4ε3 or ε4ε4. The annual rate of change reflects slope of the line in Fig. 1 and 20 years represent the value of the line (i.e., cognitive score) at 20 years. The bold values represent the three groups with the largest annual rate of change and the italicized values represent the three groups with the lowest cognitive score at 20 years.

To ensure that differences were not being driven by differences in the individual cohorts we re-ran all analysis with study cohort as a random categorical effect + (1| study). The models produced similar results in terms of effect size and significance. These findings reflect an insignificant role of the different study cohorts on the current findings. Additionally, to ensure results were not driven by differences in conversion between the groups, we reran all the models including a conversion index as an additional covariate. Conversion index was defined as 1–1/time_to_conversion, where time_to_conversion was calculated as the number of years between the current visit and the visit in which the participant had a diagnosis of cognitive decline (i.e., from NC to MCI, or from MCI to dementia). Conversion indices range between 0 and 1, with 0 indicating conversion at the current visit, and 1 indicating no conversion. These models also produced similar results to our initial findings in terms of effect size and significance suggesting that conversion differences that may be present between the groups did not influence the study findings.

Although matching samples based on follow-up year may help address potential bias in the analyses, a more conservative approach to examine bias due to attrition is to use inverse probability weighting. This method deals with attrition by giving more weight to participants who are similar to those who dropped out of the study effectively making the data more representative of the original study population. Most between group effects remained the same, therefore, we opted to report only the results which differed from the initial results. SCD-ε2 showed an increased decline in global cognition compared to NC-ε3(*t* = 7.60, *p* < 0.001) and no longer differed from NC-ε4 in global cognition. MCI-ε2 no longer exhibited a slower rate of decline than NC-ε3 in perceptual orientation but had a slower rate of change in semantic memory (*t* = -5.20, *p* < 0.001). SCD-ε4 also showed increased rate of cognitive change in perceptual orientation compared to NC-ε4 (*t* = 10.27, *p* < 0.001) and in semantic memory compared to MCI-ε4 (*t* = 4.87, *p* < 0.001).

## Discussion

While it is well-established that APOE ɛ4 positivity increases risk of AD [5, 6], few studies have examined how APOE status interacts with SCD to influence rate of cognitive change. To improve our understanding of how these two potentially independent risk factors (i.e., APOE and SCD status) interact to influence cognitive change, the current study examined the rate of cognitive change over a 27-year period in NCs, people with SCD, and people with MCI. The goal of the current study was to determine if SCD-ε2 and MCI-ε2 adults exhibit similar rates of cognitive decline to NCs, and if SCD-ε4 adults have increased rates of cognitive decline compared to NC-ε4 and MCI-ε4. In our sample of 3494 older adults, we observed that SCD-ɛ4 older adults had the largest rate of cognitive change compared to all other groups. Importantly, this rate of change in SCD-ɛ4 was also steeper than both NC-ɛ4 and MCI-ɛ4 older adults. The results also revealed that SCD-ɛ2 and MCI-ɛ2 exhibit similar rates of cognitive change to NC-ɛ3 and less change than an NC-ɛ4. The ɛ4 allele increased rate of cognitive change, particularly in people with SCD, who had the steepest rate of change. Consistent with previous findings, we did not observe differences in ɛ4 frequencies between NC and SCD [14], supporting the hypothesis that SCD and APOE ɛ4 status independently influence longitudinal cognitive decline. We did, however, observe increased frequencies of ε4 and deceased frequencies of ε3 in people with MCI compared to NCs.

People who have SCD [22] or MCI [23] have been consistently observed to exhibit increased rates of cognitive change compared to NCs. However, in the current study, both SCD-ɛ2 and MCI-ɛ2, showed similar rates of change to NC-ɛ3 and less cognitive change than NC-ɛ4. This result is consistent with previous reports indicating that the presence of the ɛ2 allele offers protective effects against risk of AD [8]. These findings indicate that even when an older adult is further along the cognitive decline trajectory (i.e., SCD or MCI), having at least one ɛ2 allele can positively influence their rate of cognitive change. On the other hand, SCD-ɛ4 older adults had increased rates of cognitive change compared to both NC-ɛ4 and MCI-ɛ4, indicating that early in the decline process, APOE status is strongly associated with rate of cognitive decline, more than in someone with MCI. These findings suggest that APOE status may be a strong risk factor for cognitive decline in people with SCD but is less strongly associated with decline once someone has cognitive impairment (i.e., MCI). This interpretation is further supported by the stronger within-group effects between APOE status and rate of cognitive change in SCD than in MCI.

Within SCD, people with an ɛ4 had steeper rates of declines than ɛ2 in all domains except perceptual speed, and steeper rates of declines than ɛ3 in all domains except perceptual orientation and perceptual speed. On the other hand, fewer effects (and smaller beta estimates) were observed for both the ɛ4:ɛ2 and ɛ4:ɛ3 differences within the MCI group. Cognitively healthy older adults with at least one ɛ4 (NC-ɛ4) also had increased rates of cognitive change compared to NC-ɛ3 and NC-ɛ2; however, as can be observed by the smaller beta estimates, the effects were less pronounced than in SCD. Overall, those with SCD-ɛ4 had the steepest rate of change indicating that in those who are cognitively unimpaired, a higher rate of cognitive impairment is associated with the ɛ4 allele [24]. These findings coincide with past research indicating that older adults with SCD who have an ɛ4 allele are at increased risk of being in the preclinical stage of AD [25]. Given that SCD may be the earliest pre-clinical stage of AD [11, 12], they are an important group to target for clinical trials and intervention strategies to reduce rate of cognitive decline before the irreversible AD-related pathology and neurodegeneration occurs. Therefore, observing that this groups’ cognition is most affected by APOE status has important implications for both research and clinical settings.

Given the multiplicative effects of SCD and APOE ɛ4 status on AD risk [14], and the fact that no cure or disease-modifying drug has become widely available for AD treatment, it is imperative to examine both risk factors (i.e., SCD and APOE) in order to better understand those at risk for the development of AD. AD pathology is thought to manifest up to two decades prior to observations of clinical symptoms [4]. Although objective decline may not be present during this stage, it is during this phase that SCD may occur [11]. Additionally, genetic testing is unlikely to occur during this stage unless there is a family history of AD. However, genetic testing may be especially important for those in the SCD stage to identify which older adults may have the highest trajectory of cognitive decline. Given that there are modifiable health and lifestyle factors that may reduce cognitive decline [26] and that APOE-specific interventions have been shown to be effective at mitigating cognitive decline in ɛ4 carriers [27], identifying these individuals at the earliest possible stage (i.e., SCD) is important.

Our findings reveal that individuals with SCD may be most impacted by the presence of at least one ɛ4 allele. This findings indicate that although ɛ4 increases risk for decline in all groups, the individuals most affected are the ones who are in the earliest stages of the disease process (i.e., those with SCD). Our findings are comparable with those of early-onset AD. For example, individuals living with early-onset AD experience greater cognitive change compared to late-onset AD [28, 29]. It is thought that these early changes result from genetic mutations in amyloid precursor protein (APP), Presenilin-1 (PSEN1), or Presenilin-2 (PSEN2) [30]. Taken together, these results suggest that it is critical to also investigate APOE in preclinical stages of the disease because it is related to increased AD risk. Specifically, genetic testing for APOE, paired with regular cognitive assessments of subjective memory complaints in cognitively normal adults may prove critical to the earliest possible treatment and/or intervention efforts. This work can aid in cohort selection for clinical trials by identifying those at greater risk even before they experience objective decline. Our work suggests that clinical trials or interventions may benefit by targeting cognitively healthy older adults who experience SCD who also have APOE ɛ4 positivity,  to better understand the risk factors associated with AD, and develop treatments for AD prevention.

There are a few strengths and limitations of the study that should be noted. Firstly, this study leverages the RUSH dataset which is a large, diverse dataset, with long follow-up durations. Long follow-up durations are important for this work because we are examining cognitive change either before (NCs) or early in the disease process (preclinical AD/SCD), and previous research has observed that the relationship between APOE ɛ4 status and cognitive decline is most pronounced after 7 years of follow-up [24]. Therefore, shorter follow-up durations may not capture the full relationship. A limitation of the current study is the comparison of APOE status to cognitive data only. Future research should examine the relationship between APOE status and structural brain changes in people with SCD using a long follow-up period. Two recent studies have examined the relationship between SCD and amyloid pathology [31] and SCD and hippocampal volume [32] which observed that amyloid pathology and hippocampal volume are important predictors of cognition in people with SCD. However, these studies used only baseline [32] or 24 month follow-ups [31]. A longitudinal design with longer follow-ups would help detect early brain biomarkers associated with disease progression in people who are ɛ4 positive. There are a number of health-related factors that are known to increase dementia risk that were not examined in this study. For example, Livingston et al., (2020) identified 12 modifiable factors that account for 40% of dementia cases. Future research should consider these factors when studying SCD. Furthermore, it should be noted that some of the APOE groups had small sample sizes (e.g., MCI-ɛ2 and SCD-ɛ2 only had 93 and 112 participants, respectively). Therefore, findings with these samples should be interpreted with caution. Future research with larger samples should also examine the interaction between rate of conversion to dementia and APOE status in these groups.

Overall, our current findings show that the relationship between the APOE ɛ4 allele and cognition is strongest in cognitively unimpaired older adults with SCD. SCD-ɛ4 older adults experience an increased rate of cognitive decline which may put them at an increased risk for future AD. These findings can improve future research and clinical trials by targeting people in the preclinical AD phase (i.e., SCD) who also possess at least one APOE ɛ4 allele. Because these older adults (SCD-ɛ4) exhibit the steepest decline in functioning, using targeted interventions for these individuals would be the most beneficial to reduce cognitive decline.

### Supplementary information

Below is the link to the electronic supplementary material.

Supplementary file1 (DOCX 15 KB)

Supplementary file2 (DOCX 518 KB)

## Data Availability

Data used in preparation of this study were obtained from the RADC Research Resource Sharing Hub, and are available from the RADC database (www.radc.rush.edu) upon registration and compliance with the data usage agreement.

## References

[1] Fleming, R., Zeisel, J. & Bennett, K. World alzheimer report 2020: Design Dignity Dementia: dementia-related design and the built environment. London, England: Alzheimer’s Disease International; 2020. p. 1.

[2] Jack CR, Bennett D, Blennow K (2018). NIA-AA Research Framework: Toward a biological definition of Alzheimer’s disease. Physiol Behav.

[3] Petersen RC. Mild cognitive impairment. CONTINUUM: Lifelong Learning in Neurology, 22(2 Dementia). 2016;404–418. 10.1212/CON.0000000000000313. 10.1212/CON.0000000000000313 PMC5390929 27042901

[4] Sperling RA, Aisen PS, Beckett LA (2011). Toward defining the preclinical stages of Alzheimer’s disease: Recommendations from the National Institute on Aging-Alzheimer’s Association workgroups on diagnostic guidelines for Alzheimer’s disease. Alzheimer’s Dement.

[5] Corder E, Saunders AM, Strittmatter WJ, Schmechel DE, Gaskell PC (1993). Gene dose of apolipoprotein E type 4 allele and the risk of Alzheimer’s disease in late onset families. Science.

[6] Heffernan AL, Chidgey C, Peng P, Masters CL, Roberts BR (2016). The Neurobiology and Age-Related Prevalence of the ε4 Allele of Apolipoprotein E in Alzheimer’s Disease Cohorts. J Mol Neurosci.

[7] Albert M, Soldan A, Gottesman R (2015). Cognitive changes preceding clinical symptom onset of mild cognitive impairment and relationship to ApoE genotype. Curr Alzheimer Res.

[8] Liu CC, Kanekiyo T, Xu HBG (2013). Apolipoprotein E and Alzheimer disease: risk, mechanisms, and therapy. Nat Rev Neurosci.

[9] Koutsodendris N, Nelson MR, Rao A, Huang Y (2021). Apolipoprotein E and Alzheimer’s Disease: Findings, Hypotheses, and Potential Mechanisms. Annu Rev Pathol Mech Dis.

[10] van Harten AC, Mielke MM, Swenson-dravis DM, Hagen CE (2018). Subjective cognitive decline and risk of MCI The Mayo Clinic Study of Aging. Published online.

[11] Jessen F, Amariglio RE, Buckley RF, van der Flier WM, Han Y, Molinuevo JL, Wagner M (2020). The characterisation of subjective cognitive decline. TheLancet Neurology.

[12] Rabin LA, Smart CM, Amariglio RE (2017). Subjective Cognitive Decline in Preclinical Alzheimer’s Disease. Annu Rev Clin Psychol.

[13] Moreno-Grau S, Rodr O, Maule A (2018). Exploring APOE genotype effects on Alzheimer’ s disease risk and amyloid b burden in individuals with subjective cognitive decline : The FundacioACE Healthy Brain Initiative ( FACEHBI ) study baseline results. Alzheimer’s Dement.

[14] Ali JI, Smart CM, Gawryluk JR (2018). Subjective Cognitive Decline and APOE e4: A Systematic Review. J Alzheimer’s Dis Published online.

[15] Barnes LL, Shah RC, Aggarwal NT, Bennett DA, Schneider JA (2012). The Minority Aging Research Study: ongoing efforts to obtain brain donation in African Americanswithout dementia. Current Alzheimer Research.

[16] Schneider JA, Aggarwal NT, Barnes L, Boyle P, Bennett DA (2009). The neuropathology of older persons with and without dementia from community versus clinic cohorts. J Alzheimer’s Dis.

[17] Bennett DA, Buchman AS, Boylea PA, Barnesa LL, Wilson RS, Schneidera JA (2018). Religious Orders Study and Rush Memory and Aging Project. J Alzheimer’s Dis.

[18] McKhann GM, Knopman DS, Chertkow H (2011). The diagnosis of dementia due to Alzheimer’s disease: Recommendations from the National Institute on Aging-Alzheimer’s Association workgroups on diagnostic guidelines for Alzheimer’s disease. Alzheimer’s Dement.

[19] Arvanitakis Z, Capuano AW, Bennett DA, Barnes LL (2018). Body Mass Index and Decline in Cognitive Function in Older Black and White Persons. J Gerontol A Biol Sci Med Sci.

[20] Lamar M, Wilson RS, Yu L, Stewart CC, Bennett DA, Boyle PA (2020). Associations of decision making abilities with blood pressure values in older adults. J Hypertens.

[21] Benjamini Y, Hochberg Y. Controlling the false discovery rate: a practical and powerful approach to multiple testing. Journal of the Royal statistical society: series B (Methodological). 1995;57(1):289-300. 10.1111/j.2517-6161.1995.tb02031.x.

[22] Morrison C, Oliver M (2022). Subjective Cognitive Decline Is Associated With Lower Baseline Cognition and Increased Rate of Cognitive Decline.

[23] Boyle PA, Wilson RS, Aggarwal NT, Tang Y, Bennett DA (2006). Mild cognitive impairment: risk of Alzheimer disease and rate of cognitive decline. Neurology.

[24] Bretsky P, Guralnik JM, Launer L, Albert M, Seeman TE (2003). The role of APOE-ε4 in longitudinal cognitive decline: MacArthur Studies of Successful Aging. Neurology.

[25] Sun Y, Yang FC, Lin CP, Han Y (2015). Biochemical and neuroimaging studies in subjective cognitive decline: progress and perspectives. CNS Neurosci Ther..

[26] Livingston G, Huntley J, Sommerlad A, Ames D, Ballard C, Banerjee S, Mukadam N (2020). Dementia prevention, intervention, and care: 2020 report of the Lancet Commission. The Lancet..

[27] Berkowitz CL, Mosconi L, Rahman A, Scheyer O, Hristov H, Isaacson RS (2018). Clinical Application of APOE in Alzheimer’s Prevention: A Precision Medicine Approach. J Prev Alzheimer’s Dis.

[28] Smits LL, Pijnenburg YAL, van der Vlies AE (2015). Early onset APOE E4-negative Alzheimer’s disease patients show faster cognitive decline on non-memory domains. Eur Neuropsychopharmacol.

[29] Tort-Merino A, Falgàs N, Allen IE (2022). Early-onset Alzheimer’s disease shows a distinct neuropsychological profile and more aggressive trajectories of cognitive decline than late-onset. Ann Clin Transl Neurol.

[30] Dai MH, Zheng H, Zeng LD, Zhang Y (2018). The genes associated with early-onset Alzheimer’s disease. Oncotarget.

[31] Hong YJ, Ho SH, Jeong JH (2023). Impacts of baseline biomarkers on cognitive trajectories in subjective cognitive decline: the CoSCo prospective cohort study. Alzheimer’s Res Ther.

[32] Caillaud M, Maltezos S, Hudon C, Mellah S, Belleville S (2023). Hippocampal Volume and Episodic Associative Memory Identify Memory Risk in Subjective Cognitive Decline Individuals in the CIMA-Q Cohort, Regardless of Cognitive Reserve Level and APOE4 Status. J Alzheimer’s Dis.

