# Correction to: The influence of APOE status on rate of cognitive decline

**DOI:** 10.1007/s11357-024-01100-8

**Published:** 2024-02-24

**Authors:** Cassandra Morrison, Michael D. Oliver, Virginia Berry, Farooq Kamal, Mahsa Dadar

**Affiliations:** 1 Department of Psychology, Carleton University, Ontario, K1S 5B6 Canada; 2 Department of Psychological Science and Neuroscience, Belmont University, Nashville, TN 37212 USA; 3 Belmont Data Collaborative, Belmont University, Nashville, TN 37212 USA; 4 Department of Psychiatry, McGill University, Montreal, QC H3A 1A1 Canada; 5 Douglas Mental Health University Institute, Montreal, QC H4H 1R3 Canada

**Correction to: GeroScience**

10.1007/s11357-024-01069-4

The article The influence of APOE status on rate of cognitive decline, written by Cassandra Morrison, Michael D. Oliver, Virginia Berry, Farooq Kamal and Mahsa Dadar, was originally published electronically on the publisher’s internet portal on 22 January 2024 without open access. With the author(s)’ decision to opt for Open Choice the copyright of the article changed on 22 January 2024 to © The Author(s) and the article is forthwith distributed under a Creative Commons Attribution 4.0 International License, which permits use, sharing, adaptation, distribution and reproduction in any medium or format, as long as you give appropriate credit to the original author(s) and the source, provide a link to the Creative Commons licence, and indicate if changes were made. The images or other third party material in this article are included in the article’s Creative Commons licence, unless indicated otherwise in a credit line to the material. If material is not included in the article’s Creative Commons licence and your intended use is not permitted by statutory regulation or exceeds the permitted use, you will need to obtain permission directly from the copyright holder. To view a copy of this licence, visit http://creativecommons.org/licenses/by/4.0/.

The original article has been corrected.

