# Supplementary material for: The influence of APOE status on rate of cognitive decline
Source: GeroScience. 2024 Jan 22;46(3):3263–74. doi: 10.1007/s11357-024-01069-4 (PMC11009190; doi:10.1007/s11357-024-01069-4)
Supplement: Supplementary file 1 — Supplementary file1 (DOCX 15 KB) [file 11357_2024_1069_MOESM1_ESM.docx]

Supplemental Table 1: Cohort information for each study as well as demographics for the participants included in the current paper.

|  | **CORE** | **LATC** | **MAP** | **MARS** | **ROS** |
| --- | --- | --- | --- | --- | --- |
| Age | 65 + | 65 + | 65 + | 65 + | 65 + |
| Cognitive Status | Cognitively healthy or dementia at baseline | Cognitively healthy or dementia at baseline | Dementia free at enrollment | Dementia free at enrollment | Dementia free at enrollment |
| Participant Recruitment | Metropolitan Chicago area and outlying suburbs | Churches, subsidized senior housing facilities, retirement communities, Latino/Hispanic clubs, organizations, and social service centers that cater to seniors in various Chicago neighborhoods and outlying suburbs | Retirement communities and subsidized senior housing throughout Chicago and Northeastern Illinois | Churches, subsidized senior housing facilities, retirement communities, African American clubs, organizations, fraternities, sororities, and social service centers in the metropolitan Chicago area and outlying suburbs. | Catholic nuns, priests, and brothers from 40 groups across the US |
| Recruitment start date | 1992 and expanded recruitment in 2008 | 2015 | 1997 and expanded recruitment in 2001 | 2004 | 1994 |
| Methods | Annual follow-up with clinical evaluation and cognitive tests | Annual follow-up with clinical evaluation and cognitive tests | Annual follow-up with clinical evaluation and cognitive tests | Annual follow-up with clinical evaluation and cognitive tests | Annual follow-up with clinical evaluation and cognitive tests |
| Number of participants | 235 | 96 | 1481 | 579 | 1105 |
| Classification  NC  SCD  MCI | 153 (65%)  58 (25%)  24 (10%) | 44 (46%)  25 (26%)  27 (28%) | 819 (55%)  345 (23%)  317 (21%) | 338 (58%)  127 (22%)  114 (20%) | 637 (58%)  221 (20%)  247 (22%) |
| APOE status  ε2  ε3  ε4 | 32 (14%)  127 (54%)  76 (32%) | 12 (13%)  68 (71%)  16 (17%) | 213 (14%)  958 (65%)  310 (21%) | 95 (16%)  294 (51%)  190 (33%) | 142 (13%)  704 (64%)  259 (23%) |
| Follow-up points in study | 1888 | 469 | 13061 | 4912 | 133392 |
| Mean follow-up time (years) | 7.69 ± 3.02 | 4.26 ± 0.92 | 8.27 ± 4.51 | 7.96 ± 4.05 | 11.5 ± 6.38 |
| Follow-up study range (years) | 0-12 | 0-5 | 0-23 | 0-17 | 0-27 |

*Notes:* NC = normal controls. MCI = mild cognitive impairment. SCD = subjective cognitive decline. CORE = Clinical CORE Study. LATC = Latino CORE Study. MAP = Memory and Aging Project. MARS = Minatory Aging Research Study. ROS = Religious Orders Study.
