# Supplementary material for: The influence of APOE status on rate of cognitive decline
Source: GeroScience. 2024 Jan 22;46(3):3263–74. doi: 10.1007/s11357-024-01069-4 (PMC11009190; doi:10.1007/s11357-024-01069-4)
Supplement: Supplementary file 2 — Supplementary file2 (DOCX 518 KB) [file 11357_2024_1069_MOESM2_ESM.docx]

Supplemental Figure 1: Longitudinal cognitive change over time by group and cognitive domain


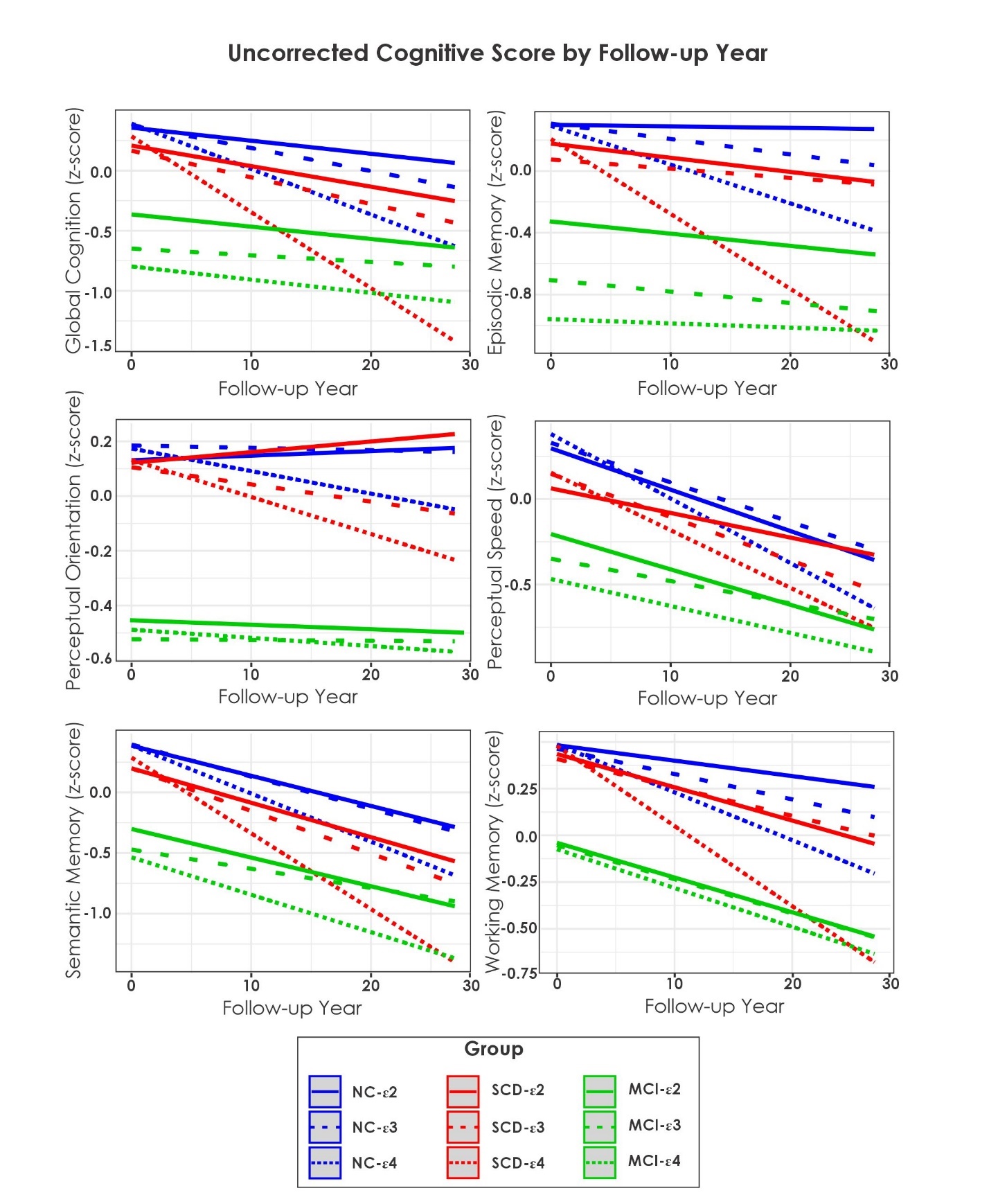


Notes: NC = cognitively normal controls. SCD = subjective cognitive decline. MCI = mild cognitive impairment
